# A pilocytic astrocytoma with novel *ATG16L1::NTRK2* fusion responsive to larotrectinib: a case report with genomic and functional analysis

**DOI:** 10.1093/oncolo/oyae254

**Published:** 2024-09-26

**Authors:** Lily Deland, Simon Keane, Thomas Olsson Bontell, Tomas Sjöberg Bexelius, Inga Gudinaviciene, Esther De La Cuesta, Francesca De Luca, Jonas A Nilsson, Helena Carén, Helena Mörse, Frida Abel

**Affiliations:** Department of Laboratory Medicine, Institute of Biomedicine, Sahlgrenska Academy, Gothenburg University, Gothenburg, Sweden; Department of Clinical Genetics and Genomics, Sahlgrenska University Hospital, Gothenburg, Sweden; Department of Laboratory Medicine, Institute of Biomedicine, Sahlgrenska Academy, Gothenburg University, Gothenburg, Sweden; Department of Clinical Pathology, Sahlgrenska University Hospital, Gothenburg, Sweden; Institute of Neuroscience and Physiology, Sahlgrenska Academy, University of Gothenburg, Gothenburg, Sweden; Section for Pediatric Oncology, Highly Specialized Pediatric Pediatrics 1, Astrid Lindgren’s Children’s Hospital, Karolinska University Hospital, Stockholm, Sweden; Department of Women’s and Children’s Health, Karolinska Institute, Stockholm, Sweden; Department of Genetics and Pathology, Laboratory Medicine Region Skåne, Lund, Sweden; Bayer Health Care Pharmaceuticals, Inc., Whippany, NJ, United States; Department of Clinical Neuroscience, Karolinska Institutet, Stockholm, Sweden; Department of Neuroradiology, Karolinska University Hospital, Stockholm, Sweden; Sahlgrenska Center for Cancer Research, Department of Surgery, Institute of Clinical Sciences, Sahlgrenska Academy, University of Gothenburg, Gothenburg, Sweden; Harry Perkins Institute of Medical Research, Perth, Australia; Sahlgrenska Center for Cancer Research, Department of Medical Biochemistry and Cell Biology, Institute of Biomedicine, Sahlgrenska Academy, University of Gothenburg, Gothenburg, Sweden; Pediatric Cancer Center, Skåne University Hospital, Lund, Sweden; Department of Laboratory Medicine, Institute of Biomedicine, Sahlgrenska Academy, Gothenburg University, Gothenburg, Sweden; Department of Clinical Genetics and Genomics, Sahlgrenska University Hospital, Gothenburg, Sweden

**Keywords:** glioma, pilocytic astrocytoma, *ATG16L1::NTRK2* fusion gene, larotrectinib

## Abstract

The outcome of pilocytic astrocytoma (PA) depends heavily on the success of surgery. In cases where surgery alone is not curative, genetic analysis can be used to identify treatment targets for precision medicine. Here, we report a pediatric PA case that underwent incomplete surgical resection due to the tumor location. Clinical routine analyses demonstrated that the tumor did not carry any *BRAF* alteration. After postoperative surveillance, according to the low-grade glioma (LGG) protocol, recurrent tumor progressions resulted in multiple chemotherapy regimens. Screening formalin-fixed paraffin-embedded tumor material using an open-ended RNA sequencing panel revealed a novel in-frame autophagy related 16 like 1-neurotrophic receptor tyrosine kinase 2 (*ATG16L1::NTRK2*) fusion gene. The *NTRK2* rearrangement was subsequently confirmed by fluorescent in situ hybridization on tumor tissue sections. Functional validation was performed by in vitro transient transfection of HEK293 cells and showed the ATG16L1::TRKB fusion protein to activate both the mitogen-activated protein kinase pathway and the phosphoinositide 3-kinase oncogenic pathways through increased phosphorylation of extracellular signal-regulated kinase, AKT, and S6. As a result of the identification of the *NTRK* fusion, the patient was enrolled in a phase I/II clinical trial of the highly selective TRK inhibitor larotrectinib. The patient responded well without significant side effects, and 8 months after the start of treatment, the contrast-enhancing tumor lesions were no longer detectable, consistent with a complete response as per Response Assessment in Neuro-Oncology (RANO) criteria. Presently, after 22 months of treatment, the patient’s complete remission is sustained. Our findings highlight the importance of screening for other oncogenic drivers in *BRAF*-negative LGGs since rare fusion genes may serve as targets for precision oncology therapy.

## Teaser

A case of a nonresectable pilocytic astrocytoma harboring a novel autophagy related 16 like 1-neurotrophic receptor tyrosine kinase 2 fusion is reported.

**Key Points**

• To the best of our knowledge, this is the first report of an autophagy related 16 like 1-neurotrophic receptor tyrosine kinase 2 (*ATG16L1::NTRK2*) fusion gene.
• Functional studies demonstrated an oncogenic effect of the *ATG16L1::NTRK2* fusion by activation of the mitogen-activated protein kinase and phosphoinositide 3-kinase pathways.
• The finding of a TRK fusion led to precision oncology by successful treatment with larotrectinib.
• Formalin-fixed paraffin-embedded tumor tissue is a viable material for detecting gene fusions by targeted RNA sequencing methods.
• The outcome of this study shows the importance of second-line screening of *BRAF*-negative low-grade gliomas by open-ended methods to increase the probability of finding targets for precision medicine.

## Introduction

Low-grade gliomas (LGGs) are the most common type of central nervous system tumors in children, and pilocytic astrocytoma (PA, WHO grade 1) is the most prevalent subtype.^1,2^ Pediatric LGG (pLGG) tumors most commonly arise in the cerebellum. They can also be found along the optic nerves, hypothalamic/chiasmatic region, cerebral hemispheres, brain stem, or spinal cord and cause significant morbidity by location and/or tumor mass.^3,4^ Gross total surgical resection is the preferred primary treatment, achieving complete remission in over one-third of all patients with pLGG.^3^ However, tumors arising in nonresectable areas, eg, the brainstem and optic nerve, result in subtotal resection and often further progression of the remnant tumor. Current treatment strategies for patients with subtotal resection include additional debulking surgeries, chemotherapy, and radiotherapy,^5^ which are known to contribute to further morbidity and mortality. The presenting symptoms and imaging findings of the patient play an important role in the management of the tumor. In 2010, the Response Assessment in Neuro-Oncology (RANO) group developed response criteria for a standardized assessment based on clinical and radiological factors.^6^ The RANO follow-up assessment includes 4 categories: progressive disease, stable disease, partial response, and complete response to treatment.

In cases with incomplete resection, understanding the molecular biology of the tumor is essential to finding strategies for targeted treatment. Previous literature has identified activation of the mitogen-activated protein kinase (MAPK) pathway as a common theme in pLGG tumors. Genetic hits of MAPK mediators occur in a mutually exclusive manner as one single hit per tumor. The *KIAA1549::BRAF* fusion gene is the most prevalent alteration in pLGG and predominantly found in PA tumors (70%-80%), for which it is often used as a diagnostic marker.^8^ Also, *BRAF*V600E mutations are fairly common, while less frequent alterations include *FGFR1/FGFR2* mutations fusions, or other *BRAF* or *RAF1* fusions.^4^ In addition, around 15% of pLGG cases are caused by familial *NF1* mutations.^8^ The unifying MAPK pathway activation in pLGG can be exploited for targeted treatment strategies in progressive and recurrent tumors. While *BRAF*V600E-driven tumors can be managed by direct inhibition of BRAF,^9,10^*BRAF* fusion-driven tumors are preferably managed by targeting mediators downstream in the MAPK pathway to avoid unwanted feedback mechanisms.^11^ Preclinical studies using MEK inhibitors such as trametinib^12^ or selumetinib^13^ show promising results by prolonged disease stability. In *BRAF*-negative pLGG, there is often involvement with other oncogenic receptor tyrosine kinases (RTK); eg, *NTRK1/2/3*, *ROS1*, *ALK*, *MET*.^4,7,14^ Agnostic treatment of RTK-fusion-driven tumors by direct receptor inhibition, eg, larotrectinib, entrectinib, and crizotinib, has shown high clinical response rates.^15^

Here, we report a case with a nonresectable PA harboring a novel autophagy related 16 like 1-neurotrophic receptor::tyrosine kinase 2 (*ATG16L1::NTRK2*) fusion gene. Upon *BRAF*-negative status by routine analysis, formalin-fixed paraffin-embedded (FFPE) tumor tissue material was screened by targeted open-end RNA sequencing, which captured the novel in-frame fusion. The patient was subsequently treated with larotrectinib, a highly selective TRK inhibitor, resulting in complete remission.

## Patient story

**Figure 1.**
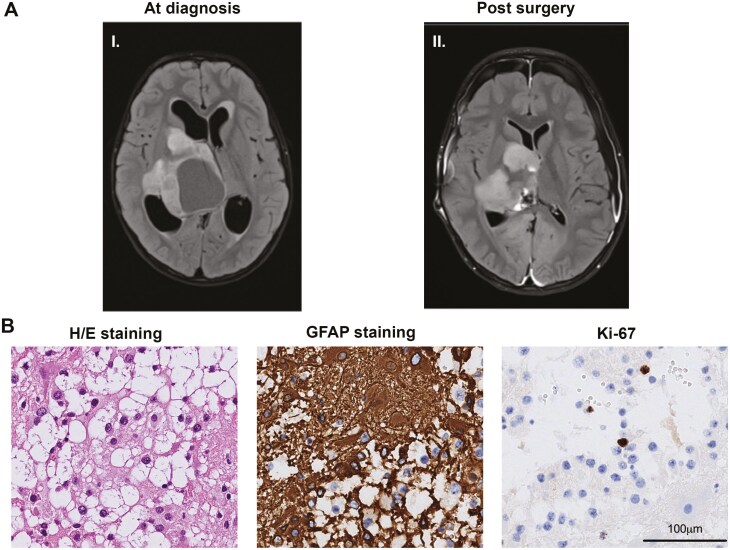
Magnetic resonance images (MRI) and histopathology. A. MRI at diagnosis (I) in September 2019, and post surgery (II). B. Hematoxylin- and eosin-stained (H/E) tumor tissue sections showed a biphasic tumor with compact and loose areas with piloid and oligodendrocyte-like cells with relatively small cell nuclei, a few Rosenthal fibers, no necrosis, and occasional mitosis. Immunohistochemistry (IHC) showed positivity for GFAP and a low Ki-67 index.

A 7-year-old boy experienced personality change, visual disturbance, and vomiting, which led to a magnetic resonance imaging (MRI) of the brain at Lund University Hospital in September 2019 (Figure 1A, I). The MRI showed a 5 × 5 × 7 cm intraventricular tumor with tumor growth also into the temporal lobe, hypothalamus, and basal ganglia. The boy underwent surgery, resulting in the resection of the intraventricular part of the tumor (Figure 1A, II). Three weeks later, he was admitted to the hospital with raised intracranial pressure due to bilateral hygroma, and a ventriculoperitoneal shunt was placed. Postoperatively, he had a loss of vision in both eyes, which was later regained. Per LGG protocol, postoperative surveillance was implemented, with MRI scans initially every 3 months. Over time, the tumor progressed. As surgery was considered too risky, chemotherapy was initiated 2 years after diagnosis with weekly vinblastine (6 mg/m²). Due to continued tumor progression, bevacizumab (10 mg/kg every 2 weeks) was added to the treatment protocol 7 months after the start of vinblastine. Despite this, there was no tumor response but increased tumor progression, and surgery was still considered perilous. A change of regimen to vincristine (1.5 mg/m² weekly) and carboplatin (550 mg/m² every 4 weeks) was implemented 9 months after the start of chemotherapy. At the MRI follow-up in the beginning of June 2022, the progression of volume and contrast enhancement of the tumor was still found in the left ventricle, with several new contrast-enhancing lesions at the right thalamus and trigone area.

Clinical routine pathological investigation of the resected tissue showed relatively small cell nuclei, few Rosenthal fibers, occasional mitosis (1 mitosis per 2 mm^2^), positivity for GFAP and synaptophysin, and a low Ki-67 proliferation rate (4.5% in hot spot areas), suggesting a LGG (Figure 1B). The tumor had normal TP53 expression, preserved expression of ATRX, and was negative for H3K27 mutation (H3 p.K28me3 assay) by immunohistochemistry (IHC), excluding other common brain tumor entities. The tumor was screened for *BRAF* fusion (Oncomine FOCUS panel, ThermoFisher Scientific), and mutations in the *BRAF* or *IDH1/2* genes (therascreen RGQ PCR, Qiagen), but no genetic alterations could be found. To confirm the histopathology diagnosis, methylation array (Infinium MethylationEPIC v1.0 BeadChip, illumina) profiling was performed on FFPE tumor tissue (tumor cell content approximately 70%; methodology as previously described^16^). Classification with the latest Molecular Neuropathology Brain classifier v.12.5 resulted in a calibrated match score of 0.99 with PA, and the same result was obtained with the clinically validated v.11b4 (score: 0.93; www.molecularneuropathology.org/mnp). The final diagnosis was *BRAF*-wildtype PA, WHO grade 1.

## Molecular tumor board

### Molecular screening of oncogenic driver

**Figure 2.**
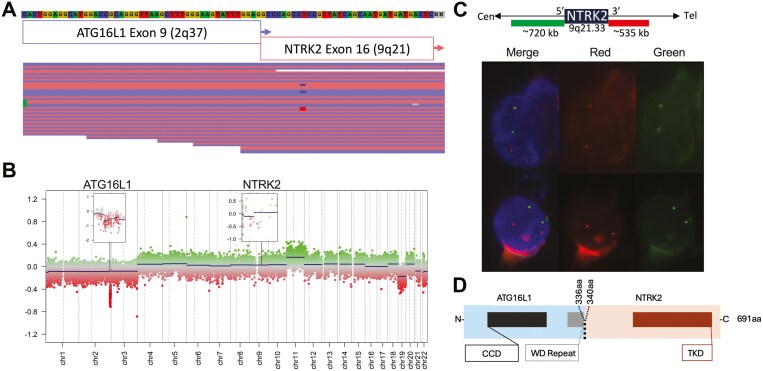
Molecular characterization of the *ATG16L1::NTRK2* fusion. (A) Targeted open-end RNA sequencing by Archer FusionPlex Pan Solid Tumor v2 panel (targeting 137 genes; eu.idtdna.com) revealed a fusion of the *ATG16L1* (exon 9, NM_017974.3) and *NTRK2* (exon 16, NM_006180.4) genes. Results were analyzed with Archer Analysis (v6.2) software using Hg19 [GRCh37] as reference for alignment and visualized in JBrowse (reads color-coded according to read orientation; method previously described^18^). The *ATG16L1::NTRK2* fusion transcript was supported by 26 unique spanning reads at the fusion point, corresponding to 60.47% of the total number of reads (*n* = 52) aligned to the position. (B) Copy number plot of chromosome 1-22 from the methylation array (EPIC v2) analysis, with zoom-in on 2q37.1 showing terminal loss within the *ATG16L1* gene, and 9q21.33 indicating imbalanced 3ʹ to 5ʹ copy numbers in the *NTRK2* gene. (C) Interphase FISH on FFPE tumor sections (4 μm) showing a split pattern in tumor cells; one or two sets of split red (3ʹ) and green (5ʹ) signals indicating an *NTRK2*-rearrangement, and one merged green/red (5ʹ/3ʹ) signal representing the *NTRK2* wild-type allele. The NTRK2 break-apart probe (ZytoLight SPEC NTRK2 Dual Color Break Apart Probe, ZytoVision GmbH, Bremerhaven, Germany) targeting 9q21.32-q21.33 is illustrated above (not to scale). One hundred interphase nuclei were counted by two independent reviewers (×50 nuclei each), and results were interpreted according to international guidelines from the European Cytogeneticists Association.^19^ Cells were counterstained with 4ʹ, 6ʹ-diamidino-2ʹ-phenylindole dihydrochloride (DAPI) and photographed at 63× as merged, red, and green channel images. (D) Schematic illustration of the ATG16L1::TRKB fusion protein showing the Coiled-Coil Domain (CCD) and WD repeat domain contributed by the N-terminal fusion partner ATG16L1 (light blue, break point at amino acid (aa) 336)) and the tyrosine kinase domain (TKD) from the C-terminal fusion partner TRKB (red, break point at aa 340) resulting in a total fusion protein length of 691 aa. The fusion break point in respective native proteins is marked (ATG16L1 NP_060444.3; TRKB NP_006171.2).

The *BRAF* negativity and well-known mutual exclusivity of MAPK alterations in PA led us to search for other oncogenic events in the current case. Since 2021, the standard procedure at Children’s Oncology units in Sweden is to perform whole-genome sequencing analysis when fresh frozen material is available.^17^ However, in the absence of that for the current case, we screened for oncogenic fusion genes using a targeted open-ended RNA sequencing strategy applicable to FFPE tumor material. The targeted RNA sequencing by Archer FusionPlex identified an *NTRK2* rearrangement joined with the novel 5' fusion partner *ATG16L1*. The genomic breakpoints were located in introns of the two genes, resulting in an *ATG16L1* (exon 9)*::NTRK2* (exon 16) in-frame fusion transcript (Figure 2A). Reinspection of the copy number alteration (CNA) plot inferred from the methylation data showed an evident loss in chromosome band 2q37.1 with a breakpoint within the *ATG16L1* gene, resulting in the loss of the 3ʹ end. No apparent CNA could be seen in chromosome band 9q21.33 (*NTRK2*). However, zooming in on the gene level of *NTRK2* hints at an copy number imbalance between the 5ʹ and 3ʹ ends, with lower levels of the 5ʹ end of *NTRK2* (Figure 2B). To confirm the results, interphase fluorescent in situ hybridization (FISH) analysis with an *NTRK2* break-apart probe was performed, verifying a DNA rearrangement of *NTRK2* (Figure 2C). The resultant ATG16L1::TRKB fusion protein and retained domains are illustrated in Figure 2D.

### Functional validation of downstream signaling pathways

**Figure 3.**
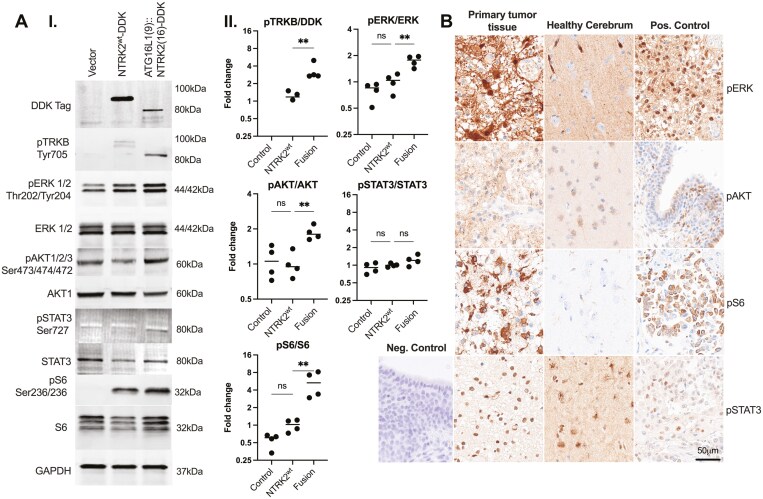
Functional analysis of MAPK, PI3K and JAK/STAT downstream signaling pathways. (A) Immunoblot result from pCMV6 vector-transfected HEK293 cells with either synthesized *ATG16L1::NTRK2* (Fusion) transcript, *NTRK2* wildtype (WT, cDNA clone from Origene, origene.com), or empty vector (Vector/Control). (I) Both *ATG16L1::NTRK2* fusion and *NTRK2*^WT^ genes were in-frame with DYKDDDDK FLAG tag (DDK Tag), resulting in 95 kDa and 80 kDa, respectively. Blots show representative bands from one of the 4 independent experiments with antibodies as follows: DDK Tag, phosphorylated (p)TRKB-Tyr705, pERK 1/2 Thr202/Tyr204 (44/42 kDa), total ERK 1/2 (44/42 kDa), pAKT 1/2/3 (60 kDa), total AKT1 (60 kDa), pSTAT3 Ser272 (80 kDa), total STAT3 (80 kDa), pS6 Ser 235/236 (32kDa), total S6 (32 kDa), and GAPDH (37 kDa). (II) Scatter plot shows the ratio pTRKB/DDK Tag, pERK/ERK, pAKT/AKT, pSTAT3/STAT3, and pS6/S6 protein quantity from the 4 experiments, calculated as fold change compared to the mean of the NTRK2^WT^ experiments. Protein quantity was normalized to stain-free protein blots (method described in previous paper^20^). Significance; *P < .05, **P < .01, ns: not significant. (B) IHC of FFPE tissue sections from the primary tumor of the *ATG16L1::NTRK2* fusion-positive case (left panel), normal healthy cerebrum (middle panel), and positive (pos) controls (right panel) stained with pERK, pAKT, pSTAT3, and pS6 antibodies. Tumor cells showed a strong nuclear and cytoplasmic immunopositivity for pERK, a positive speckled cytoplasmic pattern for pAKT, a nuclear immunopositivity for pSTAT3, and a strong cytoplasmic immunopositivity for pS6. Tissue from normal cerebral control showed a weak pERK staining of the neuropil and strong positivity in blood vessels and reactive astrocytes, pAKT showed weak neuronal cytoplasmic positive reactivity and was negative in glial cells and capillaries, pSTAT3 showed a strong positive nuclear staining, while pS6 showed a weak positive staining in some glial and nerve cells. Positive controls were as follows: a PA case with confirmed *BRAF* fusion (pERK), a normal urothelial tissue (pAKT), and a *ROS1* fusion-positive lung adenocarcinoma (pSTAT3 and pS6). Negative (neg) control with omitted primary antibody showed negative staining of normal urothelial tissue. Original magnification ×400. The scale bar represents 50 μm.

To determine the downstream signaling effect of the novel fusion gene, HEK293 cells were transiently transfected with the *ATG16L1::NTRK2* fusion, wild-type *NTRK2,* or empty vector constructs (pCMV6-Myc-DDK vector). The protein expression was subsequently quantified for the total and phosphorylated (p) protein amount for TRKB and downstream mediators of the MAPK, phosphoinositide 3-kinase (PI3K) and janus kinase (JAK)/signal transducer and activator of transcription (STAT) pathways; extracellular signal-regulated kinase (ERK), AKT, STAT3, and S6, by Western immunoblot (Figure 3A; method previously described^20^).

We observed a 2.30-fold increase in the Tyr705 phosphorylated (p) site of TRKB in the *ATG16L1::NTRK2* fusion transfected cells compared to the wild-type *NTRK2* transfected cells (Figure 3A, I). Additionally, we detected a 1.73-fold increase in pERK (Thr202/Tyr204) and a 1.85-fold increase in pAKT (Ser473) in the fusion transfection compared to the wild-type cells. As both signaling pathways of MAPK and PI3K coalesce in S6 activation, we investigated the phosphorylation of Ser235/236 in S6. We found a 4.50-fold increase in pS6 in the *ATG16L1::NTRK2* transfected cells compared to the wild-type *NTRK2* transfected cells. There was no alteration in pSTAT3/STAT3 levels between fusion and wild-type transfected cells (Figure 3A, II). Hence, we concluded that the ATG16L1::TRKB fusion protein acts primarily upon the MAPK and PI3K pathways.

The expression of pERK, pAKT, pSTAT3, and pS6 was verified in primary tumor tissue from the *ATG16L1::NTRK2* fusion-positive case. Tumor cells showed a strong immunopositivity for pERK and pS6 but were also slightly positive for pAKT and pSTAT3 (Figure 3B).

### Discussion of results and potential targeted treatment strategies

The standard of care (SOC) for PA is surgical resection, which is generally sufficient if the entirety of the tumor is removed. However, when surgical resections are incomplete, often due to tumor location, treatment of BRAF/MAPK-signaling is an option. Another alternative for second-line therapy in young children is low-dose chemotherapy to avoid radiotherapy toxicity.^5^ Also, second (and even third) surgical resections are performed in children with progressive/recurrent disease, if possible. In the current PA case, SOC resulted in subtotal resection, and additional surgery was assessed to be too risky. Moreover, since the tumor was *BRAF*-negative, no targeted treatment approaches were initially available.

Screening for other molecular tumor-driving alterations revealed a novel *ATG16L1::NTRK2* fusion, which contributed to oncogenicity by activating the MAPK and PI3K pathways. In neurotrophic tyrosine receptor kinase (*NTRK*) gene fusion events, the 5ʹ region is typically replaced by an unrelated fusion partner gene, with the 3ʹ tyrosine kinase domain (TKD) of the *NTRK* gene retained. Correspondingly, the 5ʹ partner often mediates dimerization and altered subcellular location. Similarly, the *ATG16L1:NTRK2* fusion shows a retained TKD from NTRK2 and a coiled-coiled domain from the ATG16L1 N-terminal partner which contribute to oligomerization of the protein in its native form.^21^ The constitutive activation of the TRKB tyrosine kinase domain and resultant oncogenic pathway activation may be caused by the homodimerization of the fusion protein.

Several approved treatment options exist for TRK fusion-positive tumors, eg, larotrectinib and entrectinib, which are both tumor-agnostic first-generation TRK inhibitors.^22^ Larotrectinib is a highly selective and potent inhibitor that prevents activation of the kinase domain of all tropomyosin receptor kinase (TRK) proteins (A, B, and C) and the downstream signaling of MAPK and PI3K pathways. Larotrectinib has shown promise in phase I and II clinical trials^23,24^ and is now approved in 48 countries for adult and pediatric TRK fusion-positive cancer patients.^22^ Functional verification of TRKB phosphorylation and downstream MAPK and PI3K pathway activation in the current case also allows for other targeted treatment options in the future should the patient be unresponsive to larotrectinib.

## Patient update

### Screening and enrollment in early phase clinical trial

Following the discovery of the *ATG16L1::NTRK2* fusion, the patient was referred to the early phase clinical trials unit for potential entry into the phase I/II clinical trial SCOUT (NCT02637687). The SCOUT trial is for patients up to 21 years old with tumors harboring any *NTRK* gene fusion. A minimum of at least one measurable contrast-enhancing tumor lesion by MRI is usually required to enroll a subject into clinical trials to follow the efficacy of investigational drug therapy.

**Figure 4.**
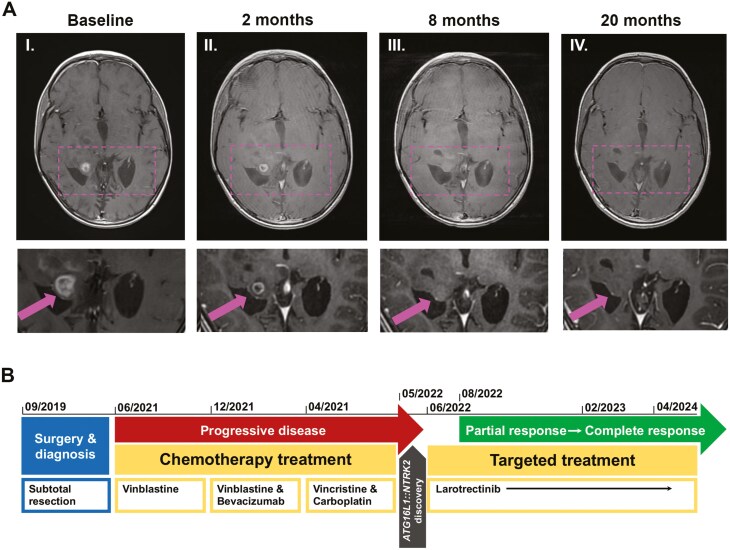
Treatment results update. (A) MRI as per RANO from baseline to last follow-up in the SCOUT clinical trial (upper panel: whole head MRI view; lower panel: zoomed in MRI view). (I) Baseline, June 2022, before the start of larotrectinib treatment, presenting with one target measurable contrast-enhancing lesion on the right thalamus (shown in the image) and 3 nonmeasurable contrast-enhancing lesions (not shown). (II) The first follow-up MRI image, August 2022, after 2 cycles of larotrectinib, showing a decrease in size of the measurable enhancing lesion ≥ 50% from the baseline MRI scan. No progression of the nonmeasurable lesions (not shown) and no new lesion occurrence was observed. The RANO imaging tumor status was a partial response. (III) In February 2023, after 8 cycles of larotrectenib, the target measurable contrast-enhancing lesion, as well as the 3 nonmeasurable contrast-enhancing lesions, were no longer detectable, and no new lesion occurrence was observed, consistent with a radiological tumor status of complete response. (IV) At the last MRI follow-up in April 2024, after 1 year and 10 months, the RANO imaging tumor status of complete response was sustained. (B) The timeline for tumor development/status and the patient’s treatment history from diagnosis in September 2019 up to the last follow-up in April 2024.

A baseline MRI scan of the brain and cervical spine using intravenous contrast injection (Gadolinium) was obtained in June 2022. One target measurable contrast-enhancing tumor lesion (target lesion, ≥10 mm × 10 mm in diameter) was identified on the right thalamus along with 3 nonmeasurable contrast-enhancing tumor lesions (Figure 4A, I). This result was assessed in a collegial discussion by radiologists to evaluate the patient’s eligibility for enrollment in the study. Based on the confirmation of *NTRK* fusion and the presence of at least one measurable contrast-enhancing tumor lesion on the MRI baseline scan, the boy was enrolled in the study in June 2022.

### Description of clinical trial and study assessments

The SCOUT trial consists of 28-day cycles with daily larotrectenib. Every other cycle, a radiological examination of tumor status with an MRI of the brain and spine was performed according to the study protocol, and the response was classified into progressive disease, stable disease, and partial or complete response as per RANO.^6^ A complete response includes the disappearance of all measurable (≥10 mm × 10 mm in diameters) and nonmeasurable (<10 mm × 10 mm in diameters) contrast-enhancing lesions, stable or improved T2-FLAIR, no occurrence of new lesions, and clinically stable or improved patient with no reliance on corticosteroids that could mask disease symptoms. Regular clinical assessments and laboratory tests were conducted before starting a new cycle throughout the study to ensure and detect any adverse events from the investigational drug larotrectenib (according to National Cancer Institute-Common Terminology Criteria for Adverse Events NCI-CTCAE version 4.03^25^). Adverse events are graded in 5 categories ranging from mild (grade 1) to death (grade 5) and were reported irrespective of association with the study treatment. For patients with LGG, the protocol requires a confirmed stable disease for at least 2 years. After this period, treatment may be suspended, but patients should continue to be monitored for tumor evaluation and safety.

### Response to targeted therapy and follow-up

Oral treatment with larotrectenib (Vitravki, BAY2757556) 100 mg BD was started in June 2022. The first MRI follow-up took place after 2 months of therapy. It showed a decrease in the size of the tumor lesion, no progression of the 3 nonmeasurable tumor lesions, stable T2-FLAIR lesions, and no new lesion occurrence. The RANO imaging tumor status was classified as a partial response (Figure 4A, II). After 8 months of larotrectenib, the target measurable contrast-enhancing lesion and the 3 nonmeasurable contrast-enhancing lesions were no longer detectable, consistent with complete response (Figure 4A, III). The complete response was sustained at the last follow-up after one year and 10 months of treatment in April 2024 (Figure 4A, IV).

Clinically, the patient has tolerated larotrectinib well without any grade 3 or 4 adverse events. He has had intermittent problems with gastrointestinal side effects, such as nausea and vomiting, but these have been manageable with medication. His WHO performance status measured by Lansky has been 90%-100% while included in the study, indicating little effect of symptoms in his daily life (as a proxy for quality of life).^26^

This case story demonstrates the feasibility and efficacy of molecular-guided therapy in refractory and unresectable tumors where SOC chemotherapy fails (Figure 4B).

## Conclusion

Detection of a novel TRK-fusion allowed the patient to be enrolled in a phase I/II trial, providing monotherapy with larotrectinib. Agnostic treatment with larotrectinib is now approved for all children with TRK fusion-positive tumors in Sweden. Consequently, due to the lack of international guidelines, we recommend screening all *BRAF*-negative pLGG tumors to reveal other oncogenic driver alterations, which may enable targeted therapy options.

## Data Availability

The data underlying this article will be shared on reasonable request to the corresponding author.
